# Reproducibility vs. Replicability: A Brief History of a Confused Terminology

**DOI:** 10.3389/fninf.2017.00076

**Published:** 2018-01-18

**Authors:** Hans E. Plesser

**Affiliations:** ^1^Faculty of Science and Technology, Norwegian University of Life Sciences, Ås, Norway; ^2^Institute for Neuroscience and Medicine (INM-6), Jülich Research Centre, Jülich, Germany

**Keywords:** computational science, repeatability, replicability, reproducibility, artifacts

A cornerstone of science is the possibility to critically assess the correctness of scientific claims made and conclusions drawn by other scientists. This requires a systematic approach to and precise description of experimental procedure and subsequent data analysis, as well as careful attention to potential sources of error, both systematic and statistic. Ideally, an experiment or analysis should be described in sufficient detail that other scientists with sufficient skills and means can follow the steps described in published work and obtain the same results within the margins of experimental error. Furthermore, where fundamental insights into nature are obtained, such as a measurement of the speed of light or the propagation of action potentials along axons, independent confirmation of the measurement or phenomenon is expected using different experimental means. In some cases, doubts about the interpretation of certain results have given rise to new branches of science, such as Schrödinger's development of the theory of first-passage times to address contradictory experimental data concerning the existence of fractional elementary charge (Schrödinger, 1915). Experimental scientists have long been aware of these issues and have developed a systematic approach over decades, well-established in the literature and as international standards.

When scientists began to use digital computers to perform simulation experiments and data analysis, such attention to experimental error took back stage. Since digital computers are exact machines, practitioners apparently assumed that results obtained by computer could be trusted, provided that the principal algorithms and methods employed were suitable to the problem at hand. Little attention was paid to the correctness of implementation, potential for error, or variation introduced by system soft- and hardware, and to how difficult it could be to actually reconstruct after some years—or even weeks—how precisely one had performed a computational experiment. Stanford geophysicist Jon Claerbout was one of the first computational scientists to address this problem (Claerbout and Karrenbach, 1992). His work was followed up by David Donoho and Victoria Stodden (Donoho et al., 2009) and introduced to a wider audience by Peng (2011).

Claerbout defined “reproducing” to mean “running the same software on the same input data and obtaining the same results” (Rougier et al., 2017), going so far as to state that “[j]udgement of the reproducibility of computationally oriented research no longer requires an expert—a clerk can do it” (Claerbout and Karrenbach, 1992). As a complement, replicating a published result is then defined to mean “writing and then running new software based on the description of a computational model or method provided in the original publication, and obtaining results that are similar enough …” (Rougier et al., 2017). I will refer to these definitions of “reproducibility” and “replicability” as *Claerbout terminology*; they have also been recommended in social, behavioral and economic sciences (Bollen et al., 2015).

Unfortunately, this use of “reproducing” and “replicating” is at odds with the terminology long established in experimental sciences. A standard textbook in analytical chemistry states (Miller and Miller, 2000, p. 6, emphasis in the original)

…modern convention makes a careful distinction between **reproducibility** and **repeatability**. …student A …would do the five replicate titrations in rapid succession …. The same set of solutions and the same glassware would be used throughout, the same temperature, humidity and other laboratory conditions would remain much the same. In such circumstances, the precision measured would be the within-run precision: this is called the **repeatability**. Suppose, however, that for some reason the titrations were performed by different staff on five different occasions in different laboratories, using different pieces of glassware and different batches of indicator …. This set of data would reflect the between-run precision of the method, i.e. its **reproducibility**.

and further on p. 95

A crucial requirement of a [collaborative test] is that it should distinguish between the repeatability standard deviation, *s*_*r*_, and the reproducibility standard deviation, *s*_*R*_. At each analyte level these are related by the equation

$$\begin{array}{l} s_{R}^{2}=s_{r}^{2}+s_{L}^{2} \end{array}$$

where $s_{L}^{2}$ is the variance due to inter-laboratory differences,.… Note that in this context reproducibility refers to errors arising in different laboratories and equipment, but using the same method: this is a more restricted definition of reproducibility than that used in other instances.

Further, the International Vocabulary of Metrology (Joint Committee for Guides in Metrology, 2006) and the corresponding standard ISO 5725-2 define as *repeatability condition of a measurement* (§2.21)

a set of conditions that includes the same measurement procedure, same operators, same measuring system, same operating conditions and same location, and replicate measurements on the same or similar objects over a short period of time

and as *reproducibility condition of a measurement* (§2.23)

a set of conditions that includes the same measurement procedure, same location, and replicate measurements on the same or similar objects over an extended period of time, but may include other conditions involving changes.

Based on these definitions, the *Association for Computing Machinery* has adopted the following definitions (Association for Computing Machinery, 2016)

**Repeatability** (Same team, same experimental setup): The measurement can be obtained with stated precision by the same team using the same measurement procedure, the same measuring system, under the same operating conditions, in the same location on multiple trials. For computational experiments, this means that a researcher can reliably repeat her own computation.

**Replicability** (Different team, same experimental setup): The measurement can be obtained with stated precision by a different team using the same measurement procedure, the same measuring system, under the same operating conditions, in the same or a different location on multiple trials. For computational experiments, this means that an independent group can obtain the same result using the author's own artifacts.

**Reproducibility** (Different team, different experimental setup): The measurement can be obtained with stated precision by a different team, a different measuring system, in a different location on multiple trials. For computational experiments, this means that an independent group can obtain the same result using artifacts which they develop completely independently.

I will refer to this definition as the *ACM terminology*. Together with some colleagues, I proposed similar definitions some years ago (Crook et al., 2013). The different terminologies are summarized in Table 1.

**Table 1:** Comparison of terminologies. See text for details.

**Goodman**	**Claerbout**	**ACM**
		Repeatability
Methods reproducibility	Reproducibility	Replicability
Results reproducibility	Replicability	Reproducibility
Inferential reproducibility		

The debate about which terminology is the proper one is heated at times, as witnessed by a discussion on “R-words” on Github (Rougier et al., 2016). One reason for the intensity of that debate may be a paper by Drummond (2009). He attempted to bring terminology in computational science in line with the experimental sciences, but at the same time argued that one should not focus on collecting computer-experimental artifacts to ensure that simulations and analyses can be re-run. While I agree with Drummond on the choice of terminology, I consider it to be essential to preserve artifacts such as software, scripts, and input data underlying computational science publications. Where re-running is successful, the published artifacts allow others to build on earlier work. Where re-running fails, which may happen due to subtle differences in system software (Glatard et al., 2015) as well as through genuine errors in problem-specific code written by researchers, well-preserved and accessible artifacts provide a basis to identify the cause of errors; Baggerly and Coombes (2009) give a high-profile example of such forensic bioinformatics.

In recent years, a number of authors have attempted to resolve this disagreement on terminology. Patil et al. (2016; see especially the Supplementary Material) give a precise definition of reproducibility, of different types of replicability, and of related terms in the form of a σ-algebra. They follow Claerbout terminology, but encounter conflicts with their own choice of terms when discussing one specific example (Patil et al., 2016; Supplementary Material, p. 6):

In this case, data and code for the original study were made available but were incomplete and/or incorrect. An independent group … examined what was provided and engineered **a new set of code which reproduced** the original results. … This differs from our definition of reproducibility because the second set of analysts … were unable to use the original code, and had to apply [modified code] instead.

Nichols et al. (2017) suggest best practices for neuroimaging based on a detailed discussion of different levels of reproducibility and replicability. They provide an informative table of which aspects of a study are fixed and which may vary at the different levels, using a terminology closer to Claerbout than to the ACM. But also these authors appear to confuse terminology slightly, since they state that “Peng reproducibility” allows for variation in code, experimenter and data analyst, while Peng's definition of reproducibility only allows for a different data analyst (Peng, 2011)—a case which Nichols et al label “Collegial analysis replicability”.

To solve the terminology confusion, Goodman et al. (2016) propose a new *lexicon for research reproducibility* with the following definitions:

- *Methods reproducibility*: provide sufficient detail about procedures and data so that the same procedures could be exactly repeated.
- *Results reproducibility*: obtain the same results from an independent study with procedures as closely matched to the original study as possible.
- *Inferential reproducibility*: draw the same conclusions from either an independent replication of a study or a reanalysis of the original study.

These definitions make explicit which aspects of trustworthiness of a study we focus on and avoid the ambiguity caused by the fact that “reproducible”, “replicable,” and “repeatable” have very similar meaning in everyday language (Goodman et al., 2016).

Applying the terminology of Goodman and colleagues to computational neuroscience, we need to consider two types of studies in particular: simulation experiments and advanced analyses of experimental data. In the latter case, we assume that the experimental data is fixed. In both types of study, methods reproducibility amounts to obtaining the same results when running the same code again; access to simulation specifications, experimental data and code is essential. Results reproducibility, on the other hand will require access to the experimental data for analysis studies, but may use different code, e.g., different analysis packages or neural simulators.

The lexicon proposed by Goodman et al. (2016) is an important step out of the terminology quagmire in which the active and fruitful debate about the trustworthiness of research has been stuck for the past decade, because it sidesteps confounding common language associations of terms by explicit labeling (explicit is better than implicit; Peters, 2004). One can only wish that it will be adopted widely so that the debate can once more focus on scientific rather than language issues.

## Author contributions

The author confirms being the sole contributor of this work and approved it for publication.

### Conflict of interest statement

The author declares that the research was conducted in the absence of any commercial or financial relationships that could be construed as a potential conflict of interest.
